# Epidemiology of Obesity and Hypertension in School Adolescents Aged 15–17 from the Region of Central Poland—A Cross-Sectional Study

**DOI:** 10.3390/ijerph18052394

**Published:** 2021-03-01

**Authors:** Piotr Wieniawski, Bożena Werner

**Affiliations:** Department of Pediatric Cardiology and General Pediatrics, Medical University of Warsaw, 63A Żwirki i Wigury Street, 02-091 Warsaw, Poland; piotr.wieniawski@wum.edu.pl

**Keywords:** obesity, hypertension, children, adolescents, primary hypertension

## Abstract

The aim of this cross-sectional study was to assess the prevalence of abnormal weight and anthropometric parameters along with abnormal blood pressure values in adolescents in Poland. Anthropometric measurements were taken in the studied age group and the correlation between these values and blood pressure values and the diagnosis of hypertension was analyzed. The main aim of the study was to characterize the particular age group in the selected population: 690 students aged 15–17 years were examined. Blood pressure and anthropometric values including height, weight, circumferences of the hips, abdomen and arms, as well as skinfolds on the back of the arm, below the scapula and the stomach, were taken. The following indexes were calculated: WHR (waist to hip ratio), WHtR (waist to height ratio), BAI (body adiposity index-hip to height ratio) and BMI (body mass index). Mean SBP (systolic blood pressure) was 112.3 (standard deviation (SD) 12.2) mmHg, and DBP (diastolic blood pressure) was 66.9 (SD 6.9) mmHg. The prevalence of hypertension in the studied group was 5.8% (3.2% boys, 2.6% girls) and prehypertension was present in 4.4% (1.6% boys, 2.8% girls). The prevalence of excess body weight was 23.6%-obesity 11.3% (40 girls, 27 boys) and overweight 12.3% (50 girls, 34 boys). Correlations between BMI and waist, hip and arm circumference, subscapular and abdominal skinfold thickness, WHtR and BAI were r = 0.86, r = 0.84, r = 0.88, r = 0.81, r = 0.75, r = 0.88 and r = 0.81, respectively (*p* < 0.05). Significant differences (*p* < 0.05) of SBP and DBP values, depending on weight category, as defined by BMI, were observed. Abnormal blood pressure values occur in one tenth and abnormal body weight in almost a quarter of the studied population. Obese and overweight children have higher SBP and DBP values compared to children with normal body weight.

## 1. Introduction

In all developed and developing countries, there is a tendency for diagnosing essential hypertension in younger children, which goes hand-in-hand with the observed parallel increase in the prevalence of overweight and obesity in the pediatric population [1,2,3,4,5,6,7,8]. It is estimated that in Europe, one in five children are overweight or obese. It is estimated that number of overweight children will rise up to 1.3 million in Europe per year, including 300,000 obese. In Poland, it will rise up to 400,000 per year, including 80,000 obese [9]. Polish epidemiological data concerning this age group are not very comparable, as they are based on different materials and different research methods. On this basis, it can be estimated only with some degree of probability that 15–30% of Polish children during adolescence are overweight or obese. Prospective studies show unequivocally that being obese at the age of 15–17 years is associated with a 17.5 times higher risk of obesity in adulthood [10]. It has been proven that 80% of obese adolescents will become obese adults and that obesity complications observed in adulthood begin to develop in childhood [11,12,13]. The main determinants of arterial hypertension in the adolescent population are the body mass index (BMI), body composition and above all, visceral obesity and the relationship between fat and lean body mass—muscle tissue.

Hypertension is recognized as one of the most important public health problems worldwide and it is a potentially reversible cause of cardiovascular disease. It is estimated that hypertension affects 4% of the pediatric population—children from 1 to 18 years of age—and the percentage of children with high normal blood pressure values is more than twice as high (9%) [14,15]. Over the last two decades, the proportions have reversed and primary hypertension is now the dominant cause of hypertension in children over 6 years of age, especially in older school children [16]. The prevalence of hypertension in adolescents aged 18 reaches about 10–11% and is approaching the incidence of hypertension in the adult population aged 18–45 (10–15%) [5,17,18,19]. This increased morbidity is explained by the worldwide obesity epidemic observed in the last decade [20], which led to identifying arterial hypertension as one of the most common health problems in the developmental age. It has been observed that body mass index (BMI) and other obesity indexes help identify children with elevated blood pressure values [21,22]. In a cross-sectional, randomized population study conducted in Poland, significant increase in the incidence of hypertension values in BMI categories from underweight to obesity was observed [18]. Fortunately, the risk of complications among those who are overweight or obese as children and those who have normal body weight as adults is the same as for those who are not overweight or obese in childhood [23]. So, fighting obesity in children and adolescents, but especially prevention, can protect them from many diseases in adulthood.

The aim of our epidemiological study was to present the risk factors described below for cardiovascular diseases as well as previously known relationships in a selected group of schoolchildren who are at the edge of adulthood. Due to little data and epidemiological studies concerning this selected age group in certain parts of the country, our main goal was to show the scale of the problem in this population. The goal of our study was to evaluate the prevalence of overweight and obesity in adolescents aged 15–17 years and to evaluate the prevalence of essential hypertension in the studied group. In the next step, our goal was to analyze anthropometric measurements in the studied age group, to analyze correlations between anthropometric measurements, between those parameters and blood pressure values and with the diagnosis of hypertension.

## 2. Materials and Methods

In total, 690 middle school and high school students aged 15–17 years who underwent screening were enrolled in our prospective study. Schools were chosen randomly. They were larger and smaller high schools and secondary schools from the region of the Masovian Voivodeship. The tests were also conducted in schools located in small villages. Sport classes were being conducted in some of those schools. No student in the studied group had been diagnosed with or suspected of hypertension before.

Nine schools were included in the study. The schools in which the study was conducted were located in Warsaw (three secondary schools) as well as in small towns (three secondary schools) and villages (three secondary schools) near Warsaw. Three schools in each of the above-mentioned categories were randomly selected. The size of the sample needed to assess the prevalence of obesity and hypertension in the group of adolescents aged 15–17 in the Polish population was calculated at a total of 500–600 adolescents with a 95% confidence level. A simple random sample of schools in the Masovian Voivodeship was selected based on a publically available list of schools. If a school administration refused to participate, a substitute school was randomly sampled from the list until nine schools were recruited in total. Participation rate was 69% (9/13). Next, random classes were selected within a school (cluster sampling). In both cases, Excel was used to assign random numbers to list items. Most of the students from the randomly selected classes took part in the study, the participation rate was above 95% and it did not differ between urban and rural schools.

Prior to the study being conducted, an informational campaign was carried out in all of the schools willing to participate. The author of the study visited each school and presented the study design to the school principal. The study had to be approved by the school principal and the class teachers. During the meetings with the parents, the class teachers presented the research project to the parents, provided them with the informational leaflets, a questionnaire and a written consent form to be filled in and signed by both students and their parents. History of risk factors of cardiovascular diseases running in the family as well as complications of hypertension was obtained from every patient’s parents or relatives.

Occurrence of obesity, hypertension, diabetes, myocardial infarction and stroke among the closest family members, parents, grandparents and siblings, was assessed. Students were asked to complete the questionnaire, developed by the authors of the study, together with their parents.

In all students, blood pressure was measured using the Korotkov method, in accordance with the standards contained in the 4th Report of the Working Group on the diagnosis, evaluation and treatment of high blood pressure in children and adolescents. Measurements were taken three times, during at least two independent visits, with an interval of at least one week. Blood pressure was measured in all schools using Riester RI-SAN spring sphygmomanometers (manufacturer: Rudolf Riester GmbH, Bruckstraße 31, D-72417 Jungingen, Germany). Measurements were taken by a physician/nurse in accordance with current standards [24,25,26]. The results were interpreted in accordance with current norms [5,24,25,26].

Arterial hypertension was diagnosed when systolic and/or diastolic blood pressure was ≥95th percentile according to norms for sex, age and/or height. All students who were diagnosed with hypertension on the basis of a screening test had oscillometric measurements taken in outpatient settings and also underwent a 24 h blood pressure measurement [27]. The results of automatic measurements using the oscillometric method were referred to the standards contained in tables and charts created during the OLAF study for the population of Polish children [28].

In all patients, the following anthropometric measurements were taken: height, weight, abdominal circumference, waist circumference and skin fold thickness.

In accordance with current recommendations, the criteria used for diagnosing hypertension in students over 16 years of age were the same as those used in the adult population [26].

In all schools, anthropometric measurements were made with aid of anthropometric measures SECA 201 ISO9001: 2000 (manufacturer: Seca Gmbh & Co. kg Hammer Steindamm 3-25 22089 Hamburg, Germany) and skinfold thickness measurements were made using the BASELINE skinfold calipers model 12-1112. The measurements were taken in accordance with the recommendations contained in the document: “Screening Test for the detection of abnormalities in physical development in children and adolescents of school age.” On the basis of the obtained measurements, the following indexes were determined: body mass index—BMI, Waist to Hip Ratio—WHR, Waist to Height Ratio—WHtR, Hip to Height Ratio—BAI (Body Adiposity Index) [29].

Skin-fold thickness was measured on the rear surface of the freely lowered arm, on the back below the inferior angle of the scapula, at the belly-midway between the umbilicus and the anterior superior iliac spine and at one-third of the distance between the anterior superior iliac spine and the pubic symphysis. Measurements of skin-fold thickness were taken in accordance with the recommendations contained in the document: “Indicators of somatic development of children and young adults in Warsaw” [30]. Obesity prevalence was estimated based on two methods. We used Polish body mass charts and BMI norms according to World Health Organization (WHO) classification to categorize participants into “normal”, “overweight”, “obese” or “underweight” [30,31]. All measurements were taken by two medical teams, with a physician and a trained nurse included in each of them. All measurements were taken using the same equipment and according to the same procedures. Technical error of measurement (TEM) was <3% both for the skinfolds’ measurements and for circumferences measurements.

For statistical analysis, R software version 3.1 was used. In the analysis, we used statistical methods such as Shapiro–Wilk test, Student’s *t*-test, Mann–Whitney U test (Wilcoxon Rank Sum Test), non-parametric chi-square test and Pearson correlation coefficient. All hypotheses were tested with a significance level of 0.05.

The study was approved by the University Bioethics Committee at the Medical University of Warsaw. Approval number of the Bioethics Committee KB/204/2009. The study was conducted in accordance with norms and standards provided in the Declaration of the World Association of Physicians from Helsinki.

The study was conducted in accordance with the World Medical Association Declaration of Helsinki.

## 3. Results

In total, 690 students aged 15–17 years were enrolled in our study, 366 girls and 324 boys, among them: 197 (28.6%) girls and 178 (25.7%) boys aged 15, 72 girls and 72 boys (equally 10.5%) aged 16 and 97 (14%) girls and 74 (10.7%) boys aged 17.

Height, weight and BMI: Average body height of 15-year-old girls in the study group was 163.2 (standard deviation (SD) 6.44) cm, and it was significantly lower than in the group of 16-year-old girls, 166 (SD 5.94) cm (*p* < 0.05). There was no difference between the group of 16-year-old girls and the group of 17-year-old girls, in which the mean height was 165.8 (SD 5.79) cm (*p* > 0.05). It has been shown that girls between 16 and 17 years do not increase in height. Average body height of 15-year-old boys was 171.7 (SD 6.84) cm and it was significantly lower than the height of boys aged 16 years (*p* < 0.05), while 16-year-old boys were shorter than the 17-year-old boys, with mean height of 175.9 (SD 6.18) cm and 178.7 (SD 6.06) cm (*p* < 0.05), respectively. Height measurement analysis showed the compliance of relevant growth quantile with growth charts for children from Warsaw, which proves that the study group is representative and implies that further conclusions concerning the analyzed age group are correct.

BMI values (kg/m^2^) ranged from 14.7 to 43.7, with an average of 21.5 (SD 3.7). In 15-year-old girls, it ranged from 15.9 to 43.7, with an average of 21.7 (SD 4.30), in girls of 16 years of age: from 15.8 to 32.3, with an average of 21.0 (SD 3.07), in the group of 17-year-old girls: 16.7–29.6, with an average of 21.1 (SD 2.74). In the group of 15-years-old boys, BMI values ranged from 14.7 to 37.5, with an average of 21.4 (SD 3.98), in 16-year-old boys—from 16.5 to 33.4, with an average 21.4 (SD 3.98), and in 17-year-old boys, BMI ranged from 17.0 to 30.4, with an average of 21.6 (SD 2.84).

**Figure 1:**
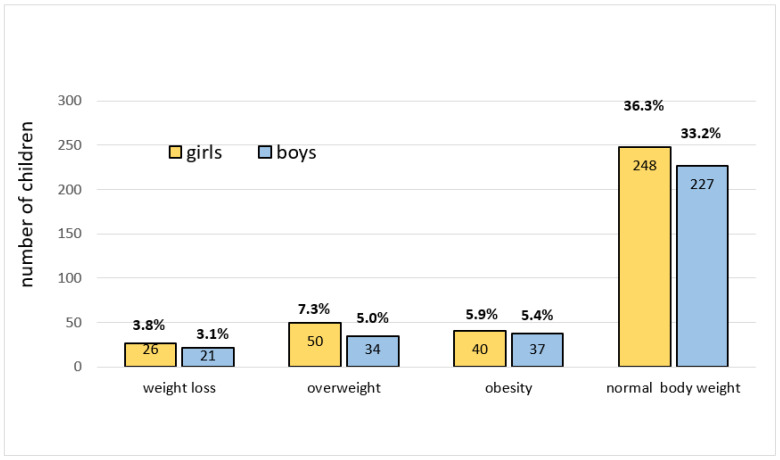
Division of studied group according to body mass index (BMI) categories.

In the whole studied group, 23.6% of children were overweight or obese, based on BMI. Obesity was diagnosed in 11.3% and overweight in 12.3% of children (Figure 1).

These percentages were also comparable among boys and girls in specified age groups. In the group of 17-year-olds, in both girls and boys, the percentage was slightly lower than in the group of 16-year-olds.

Obesity was found in 13.8% (*n* = 27) and overweight in 15.3% (*n* = 30) of girls aged 15 years. The percentage of girls who were either overweight or obese at the age of 15 was not significantly greater than the percentage of girls with obesity or overweight at the age of 16 (obesity 8.3%, overweight: 12.5%) (*p* > 0.05). The percentage of overweight or obese girls at the age of 16 was not significantly greater than in the group of 17-year-old girls, which amounted to: obesity 7.3%, overweight 11.5% (*p* > 0.05). In the group of 15-year-old boys, obesity was found in 12.5% and overweight in 10.8%. These percentages did not differ significantly from the percentages in the group of boys at the age of 16 (*p* > 0.05), which amounted to 12.7% (*n* = 9) for overweight as well as for obesity. The percentage of boys with obesity or overweight at age of 16 was not statistically significantly greater than in the group of 17-year-old boys, which amounted to 8.3% for both overweight and obesity (*p* > 0.05).

Based on the growth charts, obesity was diagnosed in 8.6% (*n* = 59) and overweight in 9.3% (*n* = 64) of the studied group. In the whole group, up to 4.8% (*n* = 33) of the girls were obese, with the same percentage being overweight (4.8% (*n* = 33)). Obese boys counted up to 3.8% (*n* = 26) of the entire group, while overweight boys counted up to 4.5% (*n* = 31) (Table 1 and Table 2).

**Figure 2:**
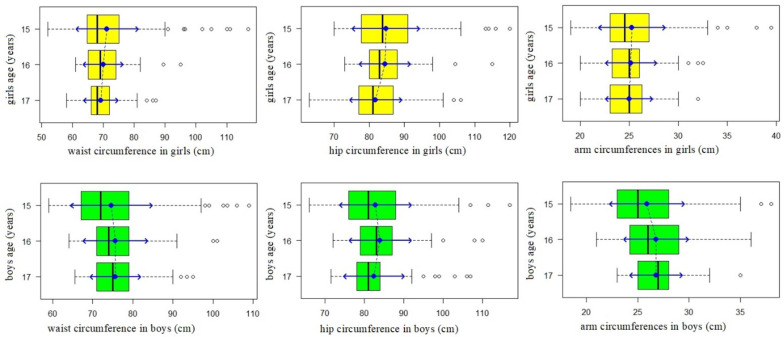
Circumferences in boys and girls in different age groups.

Circumferences and skinfolds: Analyzing the measurements of body circumferences, we found large variations in the measured parameters in the group of 15-year-old girls. Variance of waist, hip and arm circumferences in girls at the age of 15 was significantly greater than in 16-year-old girls (*p* < 0.05). It has been shown that the variance of waist, hip and arm circumferences in girls aged 16 and 17 did not differ significantly (*p* > 0.05). The greatest diversity of waist, hip and arm circumferences was observed in 15-year-old adolescents, in both girls and boys. In boys, the parameter that varied most widely was waist circumference. The variance of waist circumference in boys at age of 15 was significantly greater than in boys at the age of 16 (*p* < 0.05). Variance in waist circumference in boys at the age of 16 was also significantly greater than in 17-year-old boys (*p* < 0.05). For children aged 16 and 17, a decline in diversity of these variables was observed. No variable difference was observed in hip circumferences between a group of boys aged 15 and 16 (*p* >0.05) or between a group of 16-year-old and 17-year-old boys. Also, the variance of arm circumference in boys aged 15, 16 and 17 did not differ significantly (*p* > 0.05). Average waist circumference values in 15- and 16-year-old girls did not differ significantly and were measured up to 71.2 (SD 10) cm vs. 69.9 (SD 6.44) cm. There were no differences observed between the average waist circumference values in girls at the age of 17, 69.1 (SD 5.56) cm, and 16-year-old girls (*p* > 0.05). Average hip circumference values in 15-year-old girls in the studied group was 84.7 (SD 9.53) cm and did not differ significantly from hip circumference values in a group of 16-year-old girls, 84.5 (SD 7.26) cm (*p* > 0.05). In 17-year-old girls, average hip circumference values, 81.8 (SD 7.45) cm, were lower than in the group of girls at the age of 16 (*p* < 0.005). There were no statistically significant differences in arm circumferences in girls in the analyzed age groups. Average circumferences amounted to: 25.2 (SD 3.39) cm for 15-year-old girls, 25.1 (SD 2.61) cm for 16-year-old and 25.0 (SD 2.4) cm for 17-year-old girls (*p* > 0.05). Average waist circumference in 15-year-old boys in the studied group was 74.6 (SD 10.16) cm and did not differ significantly from the average waist circumference in a group of 16-year-old boys: 75.6 (SD 8.08) cm (*p* > 0.05). Neither was there a difference between the average waist circumference in boys at the age of 17, 75.6 (SD 6.29) cm, and boys at the age of 16 (*p* > 0.05). There were no statistically significant differences (*p* > 0.05) in hip circumferences in boys in the analyzed age groups. Average values of hip circumferences in boys at 15, 16 and 17 years amounted to (respectively): 82.8 (SD 9.11) cm, 84.0 (SD 7.91) cm and 82.4 (SD 7.73) cm. Analysis of average values of arm circumferences led to the conclusion that they are greater in 16-year-old boys than in boys of 15 years old: 26.8 (SD 3.15) cm vs 25.9 (SD 3.64) cm (*p* < 0.05). There was no difference in the average arm circumference values between the groups of boys aged 16 and 17, whose average arm circumference value equaled 26.8 (SD 2.56) cm (*p* > 0.05) (Figure 2).

**Figure 3:**
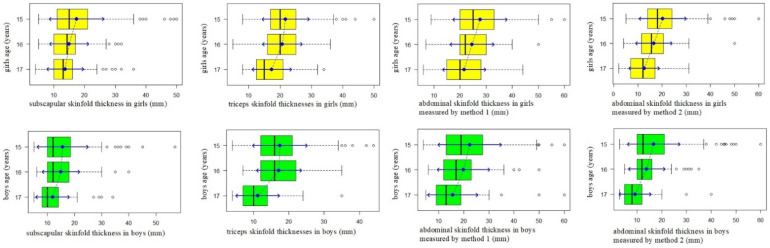
Skinfold thickness in girls and boys in different age groups.

The average thickness of the subscapular skinfold in 15-year-old girls was 17.4 (SD 8.93) mm and it was significantly greater than in girls at the age of 16: 15.0 (SD 6.4) mm (*p* < 0.05). There was no statistically significant difference (*p* > 0.05) between skinfold thickness in girls at 16 and at 17, with a mean skinfold thickness value of 13.7 (SD 6.02) mm. Conversely, in boys, there was no statistically significant difference in the average skinfold thickness values (*p* > 0.05) between 15-year-olds, 15.6 (SD 9.47 mm), and 16-year-old boys, 14.9 (SD 6.92) mm. However, significantly higher average subscapular skinfold thickness values (*p* < 0.05) were found in the group of 16-year-old boys compared to 17-year-old boys, 11.9 (SD 6.19) mm. In the case of triceps skinfold thickness, there was a tendency for its lower average values in boys as well as in girls aged 17, compared to 16-year-old adolescents of both sexes. Triceps skinfold thickness average value in 16-year-old girls was 20.7 (SD 6.34) mm and it was significantly greater than in 17-year-old girls: 17.4 (SD 6.3) mm (*p* < 0.05). In 16-year-old boys, average thickness of triceps skinfold was 17.2 (SD 6.38) mm and it was significantly greater than in 17-year-old boys: 11.2 (SD 6.19) mm (*p* < 0.05). There were no significant statistical differences between the average triceps skinfold thicknesses values neither in boys nor in girls aged 15 compared to girls and boys aged 16 (*p* > 0.05). In girls, we see a clear decrease of the average abdominal skin fold thickness with age. Accordingly, in girls at the age of 15, its average was 27.7 (SD 10.45) mm; at the age of 16, 24.6 (SD 8.48) mm, and at the age of 17, 15.6 (SD 9.99) mm. Differences in average values between adjacent age groups of girls 15 and 16 years of age and 16- and 17-years-old were statistically significant (*p* < 0.05). In 15-year-old boys, abdominal skinfold thickness was 22.4 (SD 12.65) mm, and it was not statistically significantly greater than in boys at 16: 19.9 (SD 10.41) mm (*p* > 0.05). On the contrary, 17-year-old boys’ abdominal skinfold thickness, 15.6 (SD 9.99) mm, was significantly lower than in 16-year-old boys (*p* < 0.05) (Figure 3).

**Figure 4:**
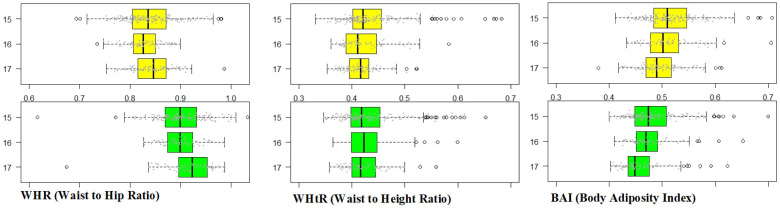
WHR, WHtR and BAI in analyzed groups. From left to right: WHR (Waist to Hip Ratio), WHtR (Waist to Height Ratio), BAI (Body Adiposity Index) in girls (yellow charts) and in boys (green charts) in the analyzed age groups.

The results of the anthropometric measurements (WHR, WHtR, BAI) were analyzed further in regard to their correlation to sex, age and other studied parameters. WHR values differed significantly for both sexes—they were higher in males and increased with age for each sex. Average WHtR values reached similar heights for both sexes regardless of age in the considered age group of 15–17 years old. BAI values varied significantly—they were higher in girls and they decreased with age for each sex (Figure 4).

Very strong correlations have been found for BMI and waist, hip and arm circumference, subscapular and abdominal skinfold thickness, WHtR and BAI—r = 0.86, r = 0.84, r = 0.88, r = 0.81, r = 0.75, r = 0.88 and r = 0.81 (*p* < 0.05). No correlation has been found for BMI and WHR. WHR was weakly positively correlated with waist circumference (r = 0.52, *p* < 0.05) and WHtR (r = 0.43, *p* < 0.05) and did not correlate or correlated very poorly with other analyzed parameters. A strong correlation has been shown between WHtR and subscapular skinfold thicknesses (r = 0.83, *p* < 0.05), abdominal skinfold thickness measured by method 1 (r = 0.77, *p* < 0.05), abdominal skinfold thickness measured by method 2 (r = 0.80, *p* < 0.05), with BAI (r = 0.81, *p* < 0.05), arm circumference (r = 0.76, *p* < 0.05), body weight (r = 0.71 *p* < 0.05), waist circumference (r = 0.92, *p* < 0.05) and hip circumference (r = 0.79, *p* < 0.05). Strong correlations have been shown for BAI and skinfold thicknesses: subscapular (r = 0.79, *p* < 0.05), triceps (r = 0.78, *p* < 0.05) abdominal circumference measured by method 1 and 2 respectively (r = 0.82 and r = 0.8, *p* < 0.05), as well as for BAI and waist circumference (r = 0.89, *p* < 0.05). Slightly weaker correlations were demonstrated for BAI and body weight (r = 0.55, *p* < 0.05), waist circumference (r = 0.66, *p* < 0.05) and arm circumference (r = 0.63, *p* < 0.05). A strong correlation was also observed for waist and hip circumference (r = 0.83, *p* < 0.05), waist and arm circumference (r = 0.84, *p* < 0.08) as well as hip and arm circumference (r = 0.76, *p* < 0.05).

Hypertension: Based on three-fold standard measurements of systolic and diastolic blood pressure, hypertension was diagnosed in 40 adolescents (5.8%) of the study population—22 boys (3.2%) and 18 girls (2.6%). The majority (32) of the 690 studied patients were diagnosed with isolated systolic hypertension, 7 patients had abnormal blood pressure values, both systolic and diastolic, and only one teenager was diagnosed with isolated diastolic hypertension. The average values of the first measurement were significantly higher than of the subsequent measurements (*p* < 0.05). Average values of the second and third measurements were similar (*p* > 0.05). The mean arithmetic value of the first and second measurements, as well as of the first and third measurements, were greater than mean values of the second and third measurements or mean of all three measurements. Based on the mean arithmetic values of the first and second measurements, hypertension was diagnosed in 49 patients (7.1%), and based on the average of the first and third measurements, it was diagnosed in 47 children (6.8%). The mean arithmetic of the first, second and third measurements and the mean of second and third measurements classify the same number of people with hypertension (5.8%).

**Figure 5:**
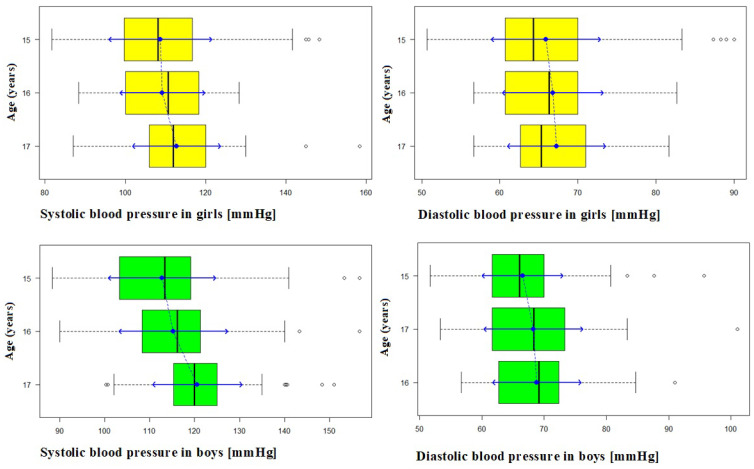
Systolic and diastolic blood pressure (BP) in girls and boys aged 15–17.

**Figure 6:**
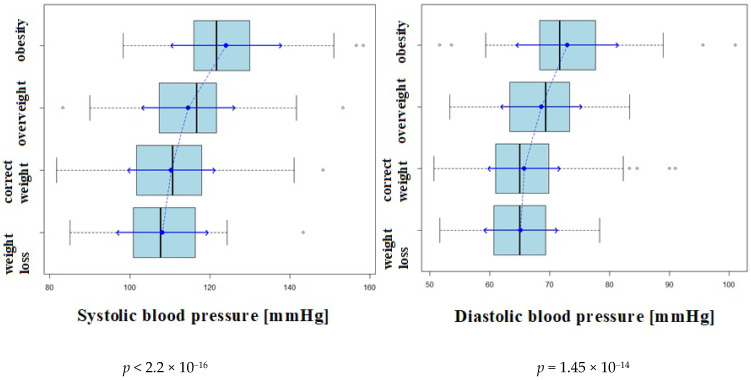
Mean systolic and diastolic blood pressure in subgroups according to BMI.

**Figure 7:**
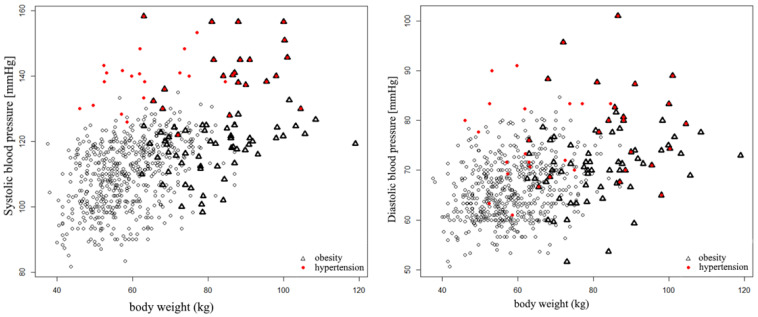
Systolic BP values according to body mass in the studied group. Triangles represent obese students, red dots represent students with hypertension.

Thereafter, the influence of selected factors on the prevalence of hypertension was evaluated. Statistically significant differences in mean values (*p* < 0.05) of both systolic and diastolic blood pressure values were observed, depending on the weight category, as defined by BMI (Figure 5, Figure 6 and Figure 7).

Figure 7 shows the relationship between body weight and blood pressure values—systolic and diastolic—separately. Additionally, by marking subjects with obesity with a triangle and those with diagnosed arterial hypertension with a red dot, the discussed relationships are now clearly presented.

Hypertension was significantly more frequent (*p* < 0.05) in adolescents who had at least one parent suffering from hypertension. Hypertension was diagnosed in 22 (10.8%) of 204 adolescents, who had at least one parent with diagnosed hypertension. Among the more than twice as large group of 464 adolescents whose parents did not suffer from hypertension, there were only 18 adolescents with diagnosed hypertension, which accounted for 3.9%.

What’s more, among the 447 adolescents who have had at least one person among their next of kin that suffered from high blood pressure, hypertension was diagnosed in 32 participants (7%), and among the 243 adolescents who did not have anyone in their family suffering from hypertension, only 5 (2%) met the criteria for the diagnosis of hypertension (*p* < 0.05).

Hypertension has been diagnosed significantly more frequently (*p* < 0.05) in adolescents who have had at least one obese parent. In the group of 144 children whose parents were obese, hypertension was diagnosed in 18 children (12.5%). In the group of 539 adolescents who did not have an obese parent, hypertension was diagnosed in 22 children, which accounted for only 4.1%.

## 4. Discussion

Our epidemiological study showed a high prevalence of overweight and obesity in the study group, that correlates with high blood pressure prevalence. Obesity has become one of the most important public health problems in many developed and developing countries [6,32,33]. Along with the increasing prevalence of obesity, higher prevalence of the comorbidities associated with obesity are observed [32]. For this reason, it is imperative that healthcare providers identify overweight and obese children so that counseling and treatment can be provided. The prevalence of overweight and obesity in children is high in most resource-rich countries worldwide [9,33]. A direct comparison of prevalence rates between countries is difficult, because of differences in definitions and measurement methods. Typically, the use of the International Obesity Task Force (IOTF) standards results in lower prevalence estimates than when other standards are used. However, studies that use comparable statistics show that rates are particularly high (greater than 30 percent) in most countries in North and South America, as well as in Great Britain, Greece, Italy, Malta, Portugal and Spain. The rates are lower in Nordic countries and the central part of Western Europe [34]. In Russia and most of the countries of Eastern Europe, the prevalence of overweight is lower (approximately 15 percent) but still increasing. The prevalence of overweight among children in China is approximately one-half of that in the United States, with numbers being substantially higher in young children than in adolescents [35]. In recent years, the percentage of obese children in developed countries has been reported to be in a plateau phase or even decreasing in number. Three large population studies that had been carried out in Poland in the last few years—depending on the used methodology—have revealed that 8–10.5–14.2% of boys were overweight respectively, and 7–6.8–6.4% of boys were obese, respectively. In girls, overweight was observed in 10.5–12–13% respectively, and obesity in 11–10–7.7%, respectively. Excess body weight is significantly higher in girls than in boys and in children living in cities rather than in those in the countryside [36,37,38,39]. However, the latest research conducted in the polish population show that high levels of economic development and urbanization have no direct impact on the prevalence of overweight and obesity in Poland [40]. As an example, BMI was compared with the percentage of fat obtained by using dual-energy X-ray absorptiometry (DXA) in a study of 979 children. The standard error for an individual BMI measurement compared to percentage of fat was 4.7 to 7.3 percent of body weight. BMI does not measure adiposity in a direct manner, and it might slightly overestimate fat percentage in short children or those who have relatively high muscle mass. BMI may also underestimate adiposity in a substantial proportion of children, such as those with reduced muscle mass due to low levels of physical activity [41]. Finally, children of different stature but the same BMI do not necessarily have the same body composition [42,43,44,45]. A discrepancy between the assessment of body mass based on the body weight growth charts and calculations based on BMI have also been observed in our study. Inconsistency of the assessment based on growth charts observed in 8.8% of children caused both over- and under-estimated results, giving, however, more often an underestimated assessment in comparison with BMI. In 29 patients (4.2%), body weight was assessed as correct using growth charts, while the assessment based on BMI showed overweight or obesity. Only in 11 patients (1.6%) did evaluation based on growth charts overestimate the assessment, indicating overweight or obesity instead of normal weight, as did BMI calculations.

In our study, a couple of methods of body composition assessment and indicators of obesity were used. BMI strongly correlates with BP values and difficult to perform anthropometric measurements, such as circumferences and skinfold thickness, which justifies its use for the assessment of overweight and obesity in daily practice.

With increasing rates of obesity in children and adolescents, an increasing number of children are being diagnosed with primary hypertension [26,46]. Hypertension in children needs to be treated in the same way as other diseases of a chronic nature. The strategy of prevention and treatment in light of available research should be long-term and focused on reducing the number of complications in adulthood. Approximately 20–30% of the global population is affected by hypertension and in most cases, it presents as essential hypertension [47,48]. Polish multi-center population-based studies have shown that hypertension affects about 30–35% of adult Poles. The incidence of hypertension increases with age. The number of adolescents with essential hypertension has rapidly increased in number. According to some authors, the problem of hypertension in older age groups may affect up to eight or even over a dozen percent of adolescents [48,49,50]. It is currently estimated that in the pediatric population, hypertension affects about 4–5% of children. According to the latest epidemiological studies, primary hypertension is its most common form, and its incidence increases with age. Prehypertension affects almost 10% of the children population [26]. The results of major studies conducted in Poland in 2007–2009, in which there were 17,000 participants aged 6.5–18.5 years, showed that the incidence rate of hypertension was 3.5%. Its findings also confirmed primary hypertension to be the most common form in children aged 10 and older. Our study has revealed that the prevalence of hypertension in adolescents aged 15–17 years from the Masovian Voivodeship is 5.8% and is slightly higher in boys—3.2% (6.8% in the boys’ group), than in girls—2.6% (4.9% in the girls’ group). These results correspond with other recent study outcomes and seem to confirm the stabilization of the growing trend, which may be related to the above-mentioned plateau phase of overweight and obesity observed in some European countries, including Poland.

Even during the developmental stage, significant damage of the arteries and left ventricular hypertrophy are observed. Overall, left ventricular hypertrophy and thickening of the intima-media complex of carotid arteries are observed in even up to 40% of children at the time of diagnosis of essential hypertension, before the introduction of any antihypertensive treatment [25,48,49,51]. For this reason, early diagnosis of hypertension, identification of accompanying risk factors and interventions that would lead to normal body weight and normotension are of the upmost importance. In the light of the above-mentioned correlations and the fact that the primary form of arterial hypertension is the dominant form of arterial hypertension among older children, it is important that doctors and those responsible for prevention and prophylaxis among adolescents promote a healthy lifestyle, physical activity and proper nutrition at every given opportunity.

Study limitations: One of the limitations of the study is the size of the group. A larger group would allow for analysis in more subgroups. Baseline skinfold caliper model 12-1112 that we used is a spring-loaded caliper with a fixed but unknown jaw pressure. Lack of its validation may be considered a limitation of the results. In the future, it should be considered to recreate the study in the same schools in the same age groups to check how the parameters have changed over time. The results of the study are used to present the epidemiological situation in a specific, selected age group of students in a specific area, and as such, they should not be generalized neither extrapolated to the entire population of young people in Poland, and even more so in other countries.

## 5. Conclusions

Abnormal blood pressure values occurred in one tenth of adolescents aged 15–17 years in the studied group in the Masovian Voivodeship. The prevalence of hypertension was 5.8% and it was slightly higher in boys (6.8%) than in girls (4.9%). The prevalence of prehypertension was 4.4% (3% boys, 5.8% girls). The prevalence of excess body weight in middle school and high school students in the studied group was 23.6%, including obesity: 11.3%, and overweight: 12.3%. Obese and overweight children presented with higher systolic and diastolic blood pressure values compared to children with normal body weight. Research methods and standards for body weight categorization based on growth charts and BMI used in the pediatric population led to slightly different epidemiological results.

BMI strongly correlated with anthropometric measurements, which justifies its use for the assessment of overweight and obesity in daily practice.

Indeed, the well-established relationship between obesity and hypertension should be demonstrated in as many epidemiological studies as possible in various age groups and in various populations. Strong correlation of the body mass category and the values of both systolic and diastolic pressure in the studied group shows how important the prevention and prophylaxis of overweight and obesity in the group of adolescents entering adulthood is.

## Figures and Tables

**Table 1:** Body mass assessment in girls according to BMI.

	Girls											
	15 Years				16 Years				17 Years			
BMI	A	B	C	D	A	B	C	D	A	B	C	D
Body Mass Charts Percentiles												
0–3	**5**	**3**							**1**	**1**		
3	**1**	**1**							**1**			
3–10	**4**	**9**			**1**	**2**			**2**	**6**		
10	**1**	**1**								**3**		
10–25	**3**	**18**			**3**	**7**				**9**		
25		**5**			**1**	**1**				**2**		
25–50		**31**	**2**			**19**			**3**	**20**		
50		**2**				**2**				**1**		
50–75		**34**	**5**			**8**				**17**	**1**	
75		**1**				**2**				**1**		
75–90		**13**	**11**	**2**		**11**	**4**	**1**		**10**	**5**	**1**
90		**1**					**2**			**1**		
90–97		**5**	**6**	**6**			**3**	**3**			**4**	**2**
97		**1**									**1**	
97–100			**6**	**19**				**2**				**4**

In the table’s cell, the number of students who met both criteria at the same time was entered: body weight category according to the percentile grids (horizontal) and BMI according to the World Health Organization (WHO) standards (vertical). BMI categories (**A**—body mass deficiency, **B**—normal weight, **C**—overweight, **D**—obesity), body mass charts categories **[0–10)**—body mass deficiency, **[10–90)**—normal weight, **[90–97)**—overweight, **[97–100]**—obesity). Green represents consistent assessments, red represents discordant assessments

**Table 2:** Body mass assessment in boys according to BMI.

	Boys											
	15 Years				16 Years				17 Years			
BMI	A	B	C	D	A	B	C	D	A	B	C	D
Body Mass Charts Percentiles												
0–3	**3**	**1**							**1**			
3						**2**						
3–10	**6**	**5**			**2**	**1**			**2**	**8**		
10	**1**	**1**			**1**	**1**			**1**			
10–25	**2**	**22**			**1**	**8**			**1**	**6**		
25		**4**										
25–50		**21**	**1**			**20**				**12**		
50		**3**				**3**						
50–75		**39**	**2**			**10**				**16**	**1**	
75		**2**				**2**						
75–90		**22**	**7**	**4**		**1**	**3**			**13**		
90			**2**									
90–97		3	**6**	**7**		**1**	**4**	**1**			**4**	**3**
97											**1**	
97–100			**1**	**11**			**2**	**8**				**3**

In the table’s cell, the number of students who met both criteria at the same time was entered: body weight category according to the percentile grids (horizontal) and BMI according to the World Health Organization (WHO) standards (vertical). BMI categories (**A**—body mass deficiency, **B**—normal weight, **C**—overweight, **D**—obesity), body mass charts categories **[0–10)**—body mass deficiency, **[10–90)**—normal weight, **[90–97)**—overweight, **[97–100]**—obesity). Green represents consistent assessments, red represents discordant assessments.

## Data Availability

Data available on request from the authors. The data that support the findings of this study are available from the first author, [piotr.wieniawski@wum.edu.pl], upon reasonable request.

## Abbreviations

BMI	body mass index
SBP	systolic blood pressure
DBP	diastolic blood pressure
BP	blood pressure
WHR	Waist to Hip Ratio
WHtR	Waist to Height Ratio
BAI	Body Adiposity Index
AHA	American Heart Association
IOTF	International Obesity Task Force
WHO	World Health Organization
TEM	technical error of measurement
